# Changes in Gut Microbiome According to Probiotic Intake in Rectal Cancer Patients Undergoing Diverting Stoma Repair: Study Protocol

**DOI:** 10.3390/jcm14207190

**Published:** 2025-10-12

**Authors:** Hyeung-min Park, Jaram Lee, Soo Young Lee, Chang Hyun Kim, Hyeong Rok Kim

**Affiliations:** Department of Surgery, Chonnam National University Hwasun Hospital and Medical School, Hwasun 58128, Republic of Korea; smart1015423@gmail.com (H.-m.P.); ramraming@naver.com (J.L.); cksantiago8@gmail.com (C.H.K.); drkhr@chonnam.ac.kr (H.R.K.)

**Keywords:** gut microbiota, probiotics, dysbiosis, rectal cancer, ileostomy repair

## Abstract

**Background**: The gut microbiome is crucial in sustaining intestinal balance and general health. Following rectal cancer surgery, the creation of a diverting stoma to protect the anastomosis results in a defunctioned colon, leading to dysbiosis. The effect of probiotic intake on gut dysbiosis following ileostomy repair remains uncertain. Thus, this study aims to determine the changes in gut microbiota based on the intake of probiotics after diverting stoma repair. **Methods**: This single-center, parallel, prospective pilot study will include patients with primary rectal cancer planning to undergo a diverting stoma during rectal cancer surgery. The study will comprise 20 patients, with 10 patients receiving synbiotics after stoma repair and 10 patients not receiving probiotics. The primary endpoint is the change in the gut microbiota of the resting colon based on the intake of probiotics, assessed through fecal testing at the following time points: before bowel resection, immediately after diverting stoma repair, and 3 weeks after diverting stoma repair. Changes in gut microbiota will be evaluated using alpha- and beta-diversity analyses based on 16S rRNA sequencing of fecal samples. **Discussion**: This study is the first prospective cohort trial investigating changes in the gut microbiota of the resting colon based on oral probiotic administration in patients undergoing diverting stoma repair. This trial is anticipated to clarify the impact of probiotic intake in these patients. **Trial registration**: Clinical Research Information Service (CRIS) of the Republic of Korea, KCT0008392, Registered on 27 April 2023.

## 1. Background

Gut microbiota represents a diverse community of microorganisms residing in the gastrointestinal tract, including viruses, bacteria, fungi, archaea, and protozoa [1,2]. Gut microbiota is essential in human health, serving immunological, metabolic, and gut-protective functions and influencing not only the intestinal system but also the pancreas, liver, and brain [2,3]. Dysbiosis, defined as an alteration in the composition or function of the gut microbiota, is implicated in the progression of numerous diseases [1,4].

In patients undergoing rectal resection for rectal cancer, a diverting stoma is often performed to reduce the risk and severity of anastomotic leakage [5,6]. During the period of maintaining the diverting stoma, the defunctioned colon experiences a deficiency in short-chain fatty acids and butyrate derived from bacteria, changes in the colonic mucosa, and a lack of oxidative substrates [7,8,9]. These changes cause gut dysbiosis characterized by a reduced diversity of bacteria, an increase in pathogenic bacteria, and a decrease in beneficial bacteria [10]. Subsequently, dysbiosis, alongside functional and structural alterations in the colon, may contribute to Clostridium difficile infection or postoperative ileus after ileostomy reversal [11,12].

Probiotics are live microorganisms aimed at promoting gut health [13]. Recent studies in colorectal cancer patients have shown that probiotics colonize the colonic mucosa, influencing the local immune responses and gut microbiota [14,15]. Additionally, probiotics alleviate postoperative symptoms and induce modification in the microbiota and inflammatory markers following surgery [16,17,18]. A recent meta-analysis reported that perioperative probiotics in colorectal cancer significantly reduced postoperative infectious complications and shortened hospital stay [19]. Furthermore, regarding dysbiosis, probiotic supplementation has been increasingly recommended as a strategy for restoring microbial balance [20,21]. However, previous studies attempted to determine the effectiveness of probiotics following stoma repair, but no significant differences were found in patients’ subjective quality of life, as assessed by questionnaires [22,23].

We hypothesized that the intake of probiotics would mitigate dysbiosis by influencing the microbiota of the defunctioned colon. However, this effect was not identified using subjective questionnaires. Therefore, this study aims to evaluate whether probiotic intake could influence gut microbiota composition and diversity following diverting ileostomy reversal by analyzing changes in the microbiota through fecal testing.

## 2. Methods

### 2.1. Trial Design

**Figure 1:**
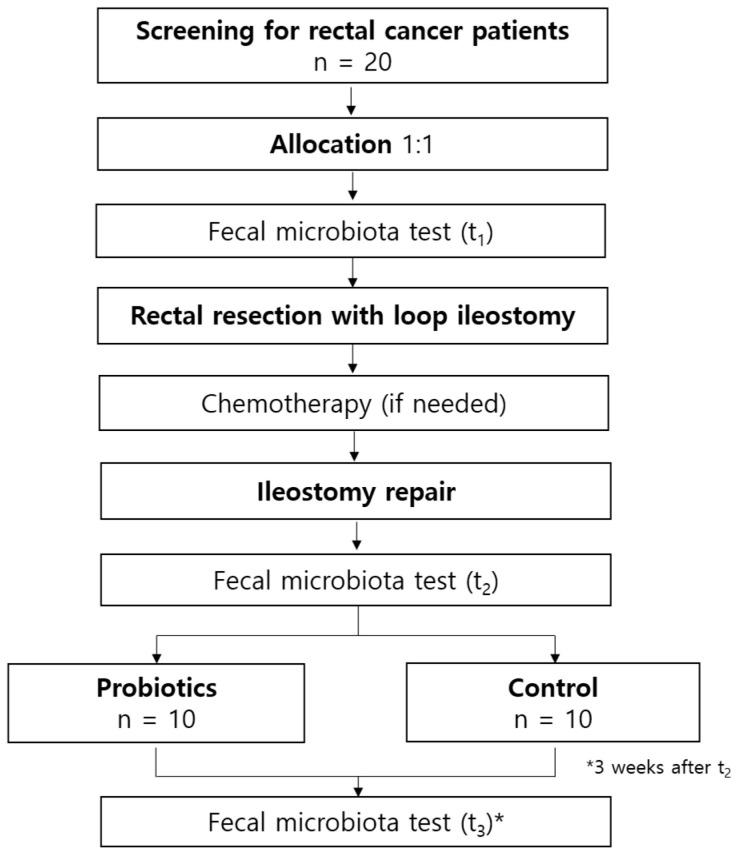
Flow diagram of the study protocol.

This single-center, parallel, prospective cohort trial with a 1:1 allocation ratio will compare patients receiving and not receiving probiotics at the time of stoma repair. This study will be assessed using the SPIRIT (Standard Protocol Items: Recommendations for Interventional Trials) checklist [24]. Figure 1 presents the study flow, including the intervention and assessment.

### 2.2. Eligibility

Patients eligible for this clinical trial will be enrolled at the Chonnam National University Hwasun Hospital in the Republic of Korea. Eligible patients include individuals with primary rectal cancer without distant metastases, those aged > 20 years, and patients who sign a consent form agreeing to enroll in this study. Exclusion criteria comprise patients requiring emergency surgery due to intestinal obstruction or perforation, those who have already undergone ostomy surgery, patients taking antibiotics for a prolonged period, and pregnant women.

### 2.3. Participants

All patients with rectal cancer who visit our outpatient clinic will be closely monitored to ensure their eligibility. Once eligibility is confirmed, the surgeon responsible for each included patient will explain this study during their outpatient clinic visit and obtain informed consent.

Given the absence of previous studies examining changes in the gut microbiota of the resting colon following ileostomy reversal, a sample size calculation was not performed. Therefore, this study was designed as a pilot trial, and we anticipated that a sample of 20 patients would be sufficient to assess the changes in microbiota based on the intake of probiotics after ileostomy repair. According to the order of patient enrollment, the first 10 consecutive patients will be assigned to the probiotic group, while the next 10 patients will be assigned to the control group. The participants will be enrolled by the surgeon at each outpatient clinic, and probiotics will be provided by a designated researcher.

At the initial outpatient visit, an assessment will be conducted to confirm eligibility based on the inclusion criteria, followed by obtaining informed consent. All patients will undergo rectal resection with loop ileostomy as previously performed. The timing of ileostomy reversal will be determined based on the necessity of chemotherapy. The first fecal test will be performed before rectal resection, and the second fecal test will be performed during the first bowel movement after ileostomy repair. Patients in the experimental group will take probiotics for 3 weeks starting from the second fecal test, whereas patients in the control group will be observed for 3 weeks without taking probiotics. Afterward, the third fecal test will be performed at the next outpatient visit. Additionally, blood tests, including complete blood count (neutrophil, monocyte, lymphocyte, hemoglobin, and platelets), albumin, and C-reactive protein, will be performed to identify changes in inflammatory markers. Table 1 demonstrated the comprehensive schedule of assessments.

### 2.4. Interventions

The synbiotic powder contains eight probiotic strains: *Bacillus subtilis* IDCC 1101, *Lactobacillus sporogenes* IDCC 1201, *Clostridium butyricum* IDCC 1301, *Lactobacillus rhamnosus* IDCC 3201, *Lactobacillus plantarum* IDCC 3501, *Bifidobacterium breve* IDCC 4401, *Bifidobacterium animalis subspecies lactis* IDCC 4301, and *Lactobacillus johnsonii* IDCC 9203 (Ildong Bioscience Co. Ltd., Pyeongtaek, Republic of Korea). Additionally, it includes prebiotics (organic galactooligosaccharide and chicory dietary fiber), vitamins, zinc, calcium, and citric acid. This multi-strain probiotic powder will be administered once daily for 3 weeks. Each sachet contains 2 g of product, and participants will take one sachet once daily for 3 weeks. Participants can withdraw from the study at any time, for any reason, without facing any consequences. If a participant meets exclusion criteria that were previously unrecognized or develops a critical medical condition that renders them ineligible, the researcher may decide to discontinue their participation in the study. We will provide sufficient synbiotics in extra amounts and remind participants several times to ensure consistent daily intake. Because antibiotics can influence the gut microbiome, we will educate participants to limit their use and monitor it at each visit. Participants will be encouraged to maintain their usual dietary habits, although no strict dietary intervention will be applied. The control group will be managed according to existing treatment protocols. We will provide appropriate medical treatment for adverse events related to the research process and compensate it according to the regulations established by our institution.

### 2.5. Outcomes

The primary outcome is changes in the gut microbiota based on the intake of probiotics at the following time points: before bowel resection, immediately after diverting stoma repair, and 3 weeks after diverting stoma repair. Genomic DNA will be extracted from bacterial samples in patients’ feces using the QIAamp DNA Stool MiniKit (Qiagen^®^, Hilden, Germany). The library will be prepared according to the 16S Metagenomic Sequencing Library Preparation Illumina Protocol. Amplification of the V3–V4 region of 16S rRNA will be performed, followed by a polymerase chain reaction. After the quality assessment of the final library, the DNA quantity will be determined using the Qubit (Thermo Fisher Scientific Inc., Waltham, MA, USA) and Agilent Bioanalyzer 1000 (Agilent, Santa Clara, CA, USA). The methods for fecal microbiota analysis will follow a previously established procedure [25]. The EZBioCloud (ChunLab, Inc., Seoul, Republic of Korea) will be used to analyze the metagenome. To evaluate within-sample richness and evenness, the Shannon and Simpson indices will be applied, while dissimilarities between samples will be assessed using the Jensen–Shannon divergence and generalized UniFrac distance. We will analyze the pattern of changes to evaluate whether a 3-week course of probiotics alleviates dysbiosis, observed after ileostomy repair, and how it differs from the gut microbiota composition identified prior to the initial surgery. Alpha- and beta-diversity analyses will be conducted, along with an assessment of the proportions of beneficial and harmful bacteria [10].

The secondary outcomes include changes in inflammatory markers and differences in gut microbiota composition based on chemotherapy. Changes in inflammatory markers will be analyzed by comparing various ratios of neutrophils, lymphocytes, albumin, and C-reactive protein. The relationship between probiotic intake and inflammatory markers will be examined to explore the potential anti-inflammatory effects of probiotics following ileostomy repair. Additionally, gut microbiota composition will be compared between patients who underwent chemotherapy before stoma repair and those who did not.

### 2.6. Blinding

Blinding of the investigators will not be required, since the trial outcomes will be based on objective quantitative data from stool and laboratory tests, mitigating any impact from unblinding. The patients will not be blinded because of the nature of the trial.

### 2.7. Data Collection and Management

All investigators will receive uniform annual training to ensure consistent assessment. A professional company performing laboratory work on fecal microbiota tests will ensure high reliability and validity. Detailed contact preferences will be collected, followed by text messages before follow-up visits to promote participant retention. A participant will be considered lost to subsequent assessment after a maximum of four contact attempts have been made.

The researcher responsible for outcome management will enter data into paper-based case report forms (CRFs). The CRF data will be meticulously reviewed for completeness and accuracy. Any discrepancies identified during the review will be resolved by the principal investigator’s verification. All CRFs will be stored in a locked, secure location accessible only to research team members. Furthermore, regular audits will be conducted to monitor access and ensure compliance with security protocols. The participant’s personal information will be protected by assigning a study identification number not associated with their identity.

The collected stool samples will be safely stored at the designated company. Upon additional request within a specified period, the following analyses can be performed and utilized in future research: calprotectin analysis using enzyme-linked immunosorbent assay, characterization of short-chain fatty acids through gas chromatography-mass spectrometry analysis, and β-glucuronidase activity analysis using fluorescence assay.

### 2.8. Statistical Methods

To compare categorical variables, the χ^2^ or Fisher’s exact test will be applied, while continuous variables will be examined by conducting Student’s *t*-test or the Mann–Whitney U-test. To evaluate statistical significance of alpha and beta diversities, the Kruskal–Wallis test and permutational multivariate analysis of variance (PERMANOVA) will be performed. A *p*-value of less than 0.05 will be considered statistically significant for all variables with. Statistical analyses will be conducted using SPSS version 27.0 (IBM Inc., Armonk, NY, USA).

### 2.9. Oversight and Monitoring

The Trial Steering Committee (TSC) will include surgeons participating in the study, acting as the authority entrusted with maintaining the scientific integrity of the trial. The TSC will convene every four weeks to verify that the study adheres to relevant principles and to offer comprehensive oversight. Day-to-day support will be ensured by the principal investigator, who will supervise the trial, manage participant enrolment, and assume clinical responsibility for patients, alongside the trial coordinators, who will collect data and follow up with patients. The day-to-day support team will meet weekly.

The entire data collection and management process will be monitored by TSC independently of the sponsor and any competing interests. Adverse events will be identified and recorded from the time a participant signs the consent form until the last fecal testing on the 21st postoperative day. The investigator will be responsible for assessing each adverse event, and any life-threatening events will be addressed within 24 h of identification. The institutional review board of the Chonnam National University Hwasun Hospital will review the clinical trial at least annually, including consent forms, protocol compliance, planned procedures, and data quality in CRFs. If protocol revisions are essential during a clinical trial, amendments will be made with the consent of the investigators and study participants. Then, the institutional review board will discuss and endorse the revised protocol.

### 2.10. Dissemination Plans

The findings of this clinical trial will be presented through posters or oral presentations at relevant scientific meetings and shared in a journal publication. The results will be disclosed irrespective of the size or nature of the effect.

## 3. Discussion

Dysbiosis is linked to numerous diseases, including inflammatory bowel diseases [26], autoimmune disorders [27], metabolic diseases [28], and neurologic disorders [29]. Moreover, dysbiosis may initiate necrotizing enterocolitis in newborns [30], as well as colorectal cancer and *Clostridium difficile*-associated diarrhea in adults [4,31]. Gut microbiota modulation aiming to alleviate dysbiosis may represent prevention and treatment strategies for the above-mentioned diseases.

The impact of probiotics on the gut microbiota has been investigated. Previous studies in patients who received probiotics after surgery reported an increase in beneficial bacteria and a reduction in harmful bacteria within fecal microbiota [17,32]. Alander et al. [33] showed that *lactobacilli* became predominant, with increased aerobic and anaerobic bacteria in stool samples after a 10-day treatment with *Lactobacillus* GG. Similarly, another study investigating treatment with *L. rhamnosus* showed an increased frequency and number of *Enterococcus* strains [34]. Thus, probiotics could modify the gut microbiome, potentially influencing gut dysbiosis. Recent studies have indicated that probiotics may help correct dysbiosis or mitigate its severity [35,36]. Therefore, administering probiotics to manage gut dysbiosis would be a worthwhile approach.

Several studies reported the effects of probiotics following ileostomy reversal. Two double-blind, placebo-controlled, randomized trials that used questionnaires showed no significant difference in the overall quality of life score [22,23]. Additionally, a study investigating patients scheduled for ileostomy repair with confirmed diversion colitis compared colonic inflammation before and after ileostomy repair using colonoscopic biopsy [37]. The results demonstrated that probiotics reduced postoperative colitis and short-term symptoms in patients. However, in that study, probiotics were infused into the efferent stoma loop with 250 mL of 0.9% physiological saline every two days, starting 20 days before repair surgery, instead of oral administration. Thus, determining the effectiveness of oral probiotic administration from the results was invalid owing to the design of this study.

In addition to temporary diverting stomas, perineal colostomy has been introduced as an alternative reconstructive method in colorectal surgery. By relocating the stoma to the perineum, this technique may improve body image, reduce the risk of parastomal hernia, and offer better cosmetic outcomes [38]. Nevertheless, the approach remains technically demanding, carries the risk of local complications, and its long-term functional results are not yet well established. While our study specifically focuses on microbiota changes after diverting stoma repair, the concept of microbial modulation through probiotics may also be relevant to patients undergoing alternative procedures such as perineal colostomy, thereby broadening the potential applicability across reconstructive strategies.

Recent evidence has also highlighted the role of butyrylcholinesterase as a novel biomarker in colorectal surgery. Low perioperative or postoperative butyrylcholinesterase levels have been associated with impaired nutritional status, systemic inflammation, and an increased risk of complications such as surgical-site infection and delayed recovery [39]. Because it can be measured simply and inexpensively, butyrylcholinesterase provides clinicians with a practical tool for risk assessment. When combined with microbiota-targeted interventions like probiotics, such biomarkers may contribute to a more integrated and personalized approach to improving postoperative outcomes in colorectal cancer patients.

A limitation of this protocol is that participants are allocated sequentially rather than randomized. While this method is pragmatic for a small, single-center pilot trial, it carries the potential for selection bias and confounding, which should be acknowledged when interpreting the findings. A further limitation is that the intervention is a commercially available multi-component synbiotic product containing not only probiotics but also prebiotics, vitamins, and minerals. Therefore, the effects cannot be attributed exclusively to probiotics; however, this formulation was chosen because it reflects actual clinical practice and has been utilized in previous clinical studies. In addition, the absence of placebo and blinding may introduce additional bias despite the use of objective outcome measures. This limitation reflects the exploratory design of this protocol and indicates that future randomized, placebo-controlled trials will be essential to validate and expand upon our findings. Finally, dietary intake was not strictly controlled, which may have influenced gut microbiota composition despite advising participants to maintain their usual habits.

To our knowledge, this is the first prospective cohort trial to investigate changes in gut microbiota based on oral probiotic administration in patients undergoing diverting stoma repair. The findings of this trial may offer a theoretical basis for understanding the effects of probiotics on dysbiosis during diverting stoma repair. Moreover, the results may offer insights that can inform perioperative management strategies and contribute to developing microbiota-targeted interventions to improve postoperative recovery in colorectal cancer patients.

## 4. Trial Status

This trial is currently open for recruitment. The current study protocol version is 1.3, which was approved on 21 March 2024. The recruitment began on 10 May 2023, and we anticipate reaching the maximum number of included patients by December 2025. As of 28 August 2025, 70% of participants have been enrolled, with final fecal samples collected from 55% of participants.

## Figures and Tables

**Table 1:** Schedule of assessments, interventions, and follow-up.

	Study Period			
	Enrolment	Post-Allocation		Close-Out
Time Point	t1	t2 (Before Rectal Resection)	t3 (After Ileostomy Repair)	t4 (Outpatient Visit)
Enrolment:				
Eligibility screen	X			
Informed consent	X			
Allocation	X			
Interventions:				
Probiotic intake			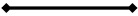	
Standard care			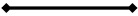	
Assessments:				
Fecal testing		X	X	X
Serum laboratory test *	X		X	X

* Complete blood count (neutrophil, monocyte, lymphocyte, hemoglobin, platelets), albumin, and C-reactive protein.

## Data Availability

The data generated from this study will be made available by the corresponding author upon reasonable request and after consultation among the authors, subject to ethical and legal restrictions related to participant confidentiality and privacy.

## Abbreviations

The following abbreviations are used in this manuscript:

CRF	Case Report Form
TSC	Trial Steering Committee
